# Diffusion mobility increases linearly on liquid binodals above triple point

**DOI:** 10.1038/s41598-022-26390-w

**Published:** 2023-02-16

**Authors:** Nikita A. Dmitryuk, Lucia A. Mistryukova, Nikita P. Kryuchkov, Sergey A. Khrapak, Stanislav O. Yurchenko

**Affiliations:** Bauman Moscow State Technical University, 2nd Baumanskaya Street 5, Moscow, Russia 105005

**Keywords:** Phase transitions and critical phenomena, Condensed-matter physics, Nonlinear phenomena

## Abstract

Self-diffusion in fluids has been thoroughly studied numerically, but even for simple liquids just a few scaling relationships are known. Relations between diffusion, excitation spectra, and character of the interparticle interactions remain poorly understood. Here, we show that diffusion mobility of particles in simple fluids increases linearly on the liquid branch of the liquid–gas binodal, from the triple point almost up to the critical point. With molecular dynamics simulations, we considered bulk systems of particles interacting via a generalised Lennard–Jones potential, as well as ethane. Using a two-oscillator model for the analysis of excitations, we observed that the mobility (inverse diffusion) coefficient on the liquid–gas binodal increases linearly above the triple point until the dispersion of high-frequency spectra has a solid-like (oscillating) shape. In terms of a separate mode analysis (of longitudinal and transverse modes), this corresponds to crossed modes in the intermediate range of wavenumbers *q*, between the hydrodynamic regime (small *q*) and the regime of individual particle motion (large *q*). The results should be interesting for a broad community in physics and chemistry of fluids, since self-diffusion is among the most fundamental transport phenomena, important for prospective chemical technologies, micro-, nanofluidics, and biotechnologies.

## Introduction

Diffusion plays an important role in various transfer processes ranging from science and technology to multi-agent and social behaviour in wildlife. It plays a decisive role in biological processes^1,2^, as well as in mechanisms and kinetics of chemical reactions. Knowledge of the mechanisms governing diffusion provides opportunities for prospective biotechnologies, chemical, and pharm-industries^3^. Diffusion has been studied in details in solid systems^4^, because of its practical importance in metallurgy for alloying^5–7^ and operation of semiconductor electronics^8,9^. Diffusion processes in gases are also rather well understood.

However, understanding the diffusion process in liquids remains rather limited and fragmented, although some progress has been achieved over the years^10–15^. A few scalings and relationships are known for approximate description of diffusion in different systems with varying accuracy. Perhaps, the simplest of them is the Arrhenius law^16^, widely used to estimate the temperature dependence of the diffusion coefficient in liquids. However, this approximation is unsuitable for an accurate determination of the diffusion coefficient over a wide temperature range, because of neglecting the effects of collective dynamics in liquids, such as dynamic viscosity. Other useful relationships for diffusion in liquids include the excess entropy scaling of transport coefficients^17–20^, their freezing temperature and density scalings^21–25^, and the Stokes-Einstein relation between the diffusion and shear viscosity coefficients^26–31^. Technically, extensive results from numerical simulations^32–34^ and machine learning^35^ are applied to study diffusion across coupling regimes. However, interrelations between diffusion, excitation spectra, and specific form of the interaction potential between the particles have never been studied in sufficient detail in a broad liquid regime, to the best of our knowledge.

In this paper, we studied classical fluids interacting via generalised Lennard–Jones potentials. With molecular dynamics (MD) simulations, we studied generalised Lennard–Jones systems with varied long-range attraction. We discovered that the particle mobility along the liquid branch of the liquid–gas binoidal increases linearly with the temperature, from the triple point almost up to the critical point. We found that the particle mobility increases linearly in the regime where the spectra of high-frequency collective excitations calculated with the two-oscillator model have a solid-like (oscillating) shape. In terms of the separate mode analysis, this corresponds to the situation with intersecting dispersion relations of the longitudinal and transverse excitations. This intersection occurs at intermediate wavelengths, between the hydrodynamic regime (small wavenumbers *q*) and the regime of individual particle motion (large wavenumbers *q*). The discovered linear dependence of the particle mobility on temperature and its relation to the excitation spectra are inherent for all studied systems, illustrating a novel kind of universal relationship for diffusion in liquids. Our results prove unambiguously that diffusion in the liquid state is closely related to the properties of collective excitations.

**Figure 1:**
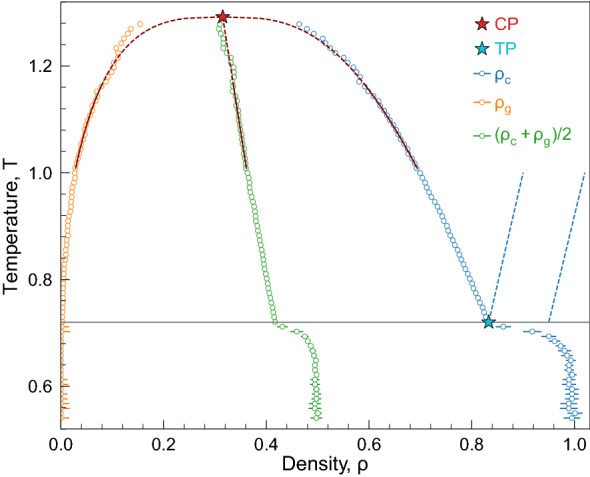
Gas-liquid binodal for the LJ12-6 system: The orange and blue symbols are densities of gas and condensate obtained through fitting MD data by Eq. (6). The green symbols are median $\rho _m=(\rho _g+\rho _c)/2$. The solid red line is a fit by Eq. (7). The triple and critical points (CP and TP, respectively) are indicated by red and blue stars, respectively.

## Results and discussion

### Particle mobility

We analyzed the transport properties and their relationship with collective modes on liquid binodals for systems interacting via a generalised Lennard–Jones (LJ*n*-*m*) potential:

1 $$\begin{aligned} U_{n-m}(r)=4 \varepsilon \left[ \left( \frac{\sigma }{r}\right) ^{n}-\left( \frac{\sigma }{r}\right) ^{m}\right] , \end{aligned}$$

where $\varepsilon$ and $\sigma$ are the characteristic energy and length scales, respectively. The reduced units of temperature $T/ \varepsilon \rightarrow T$, distance $r/ \sigma \rightarrow r$, and density $\rho \sigma ^ 3 \rightarrow n$ are used throughout the paper.

We considered LJ12-4, LJ12-5, LJ12-6, and LJ16-6 potentials. We also simulated ethane^36^ to compare the obtained results for the spherically symmetric LJ*n*-*m* systems with those for a system with a non-spherical interaction. In the chosen model, ethane molecule is considered as a pair of hardbound $\textrm{CH}_3$ radicals interacting with radicals of other molecules through the potential^36^:

2 $$\begin{aligned} U_{{\textrm{ethane}}}(r) = {\tilde{\varepsilon }}\left[ \left( \frac{\sigma }{r}\right) ^{16}-\left( \frac{\sigma }{r}\right) ^{6}\right] , \end{aligned}$$

where ${\tilde{\varepsilon }} = 0.69396$ kcal/mol and $\sigma = 3.783$Å. Details of simulations are provided in Methods.

An example of the obtained gas-liquid binoidal for the LJ12-6 system and near-critical approximation of Eq. (7) are shown in Fig. 1. Note that there is an apparent kink on the condensed binodal at $T\simeq 0.72$ in Fig. 1, which indicates density drop at melting and corresponds to the triple point position. Very similar behaviour to that shown in Fig. 1 is inherent to other systems we studied and agrees with recent results reported in Ref.^37^. The densities and temperatures of the critical and triple points, as well as the obtained parameters of the fit (7) are summarised in Table 1. Here, temperatures and densities are given in reduced units for LJ systems and in K and $\text {kg}/\text {m}^3$ for ethane, respectively. The parameters of critical and triple points for ethane are taken from Ref.^38^.

**Table 1:** The values of densities and temperatures of critical and triple points and parameters of fits by Eq. (7) for considered models.

LJn-m	$T_{\textrm{CP}}$	$\rho _{\textrm{CP}}$	$T_{\textrm{TP}}$	$\rho _{\textrm{TP}}$	*A*	*a*
LJ12-4	4.85	0.291	1.675	0.959	0.559	0.107
LJ12-5	2.18	0.304	0.985	0.877	0.804	0.208
LJ12-6	1.29	0.315	0.687	0.847	1.002	0.326
LJ16-6	1.55	0.316	0.934	0.828	0.969	0.334
Ethane	305.3	206.7	90.34	651.9	113.1	1.158

After the gas-liquid binoidal was obtained, we calculated the particle mobility at densities and temperatures of liquid coexisting with the gaseous phase (at the liquid branch of the binodal). The self-diffusion coefficient *D* was determined from the long-time mean-square deviation of particles:

3 $$\begin{aligned} \begin{aligned} \sigma ^2(t)&= N^{-1}\sum \limits _{\alpha = 1}^{N} (r_{\alpha }(t) - r_{\alpha }(0))^2, \\ D&= \lim _{t\rightarrow \infty }{\sigma ^2(t)/6t}, \end{aligned} \end{aligned}$$

where $\sigma ^2$ is the standard deviation, and *t* is time. The particle mobility $\mu$ is related to the diffusion coefficient via the Einstein relationship

4 $$\begin{aligned} \mu = {D}/{T}, \end{aligned}$$

where *T* is the temperature.

**Table 2:** Particle mobility calculated for studied generalised LJ systems (in normalised units) and for ethane (in *K* and $nm^2 / (\mu s \cdot K)$ and ${\tilde{\mu }}=\mu \sqrt{\varepsilon M/\sigma ^2}$, where $M=30.07\,g/mol$ is ethane molar mass).

**LJ12-4**														
*T*	1.788	2.012	2.247	2.463	2.687	2.912	3.137	3.318	3.412	3.507	3.601	3.696	3.790	3.884
$\rho$	0.944	0.914	0.882	0.850	0.819	0.783	0.749	0.719	0.703	0.687	0.670	0.653	0.635	0.617
$\mu$	0.031	0.039	0.048	0.056	0.065	0.075	0.085	0.093	0.097	0.103	0.108	0.113	0.119	0.124
*T*	4.073	4.168	4.262	4.309	4.368	4.426	4.485	4.544	4.602	4.661	4.720	4.778	4.837	–
$\rho$	0.578	0.558	0.536	0.525	0.510	0.495	0.479	0.461	0.443	0.422	0.398	0.369	0.322	–
$\mu$	0.137	0.144	0.154	0.159	0.163	0.172	0.178	0.186	0.198	0.208	0.223	0.246	0.286	–
**LJ12-5**														
*T*	1.032	1.160	1.288	1.416	1.544	1.672	1.704	1.739	1.773	1.808	1.842	1.877	1.911	1.946
$\rho$	0.867	0.833	0.797	0.761	0.723	0.680	0.670	0.658	0.647	0.634	0.622	0.608	0.594	0.579
$\mu$	0.041	0.054	0.068	0.082	0.096	0.115	0.119	0.124	0.130	0.134	0.142	0.147	0.152	0.161
*T*	2.00	2.026	2.049	2.071	2.094	2.117	2.140	2.162	–	–	–	–	–	–
$\rho$	0.552	0.539	0.525	0.511	0.494	0.475	0.451	0.416	–	–	–	–	–	–
$\mu$	0.175	0.181	0.191	0.198	0.208	0.221	0.238	0.269	–	–	–	–	–	–
**LJ12-6**														
*T*	0.721	0.748	0.774	0.801	0.829	0.856	0.882	0.909	0.936	0.964	0.991	1.008	1.024	1.039
$\rho$	0.832	0.821	0.808	0.796	0.784	0.772	0.758	0.745	0.733	0.717	0.704	0.694	0.685	0.676
$\mu$	0.053	0.058	0.064	0.070	0.076	0.081	0.089	0.095	0.100	0.109	0.116	0.121	0.126	0.130
*T*	1.070	1.085	1.100	1.116	1.131	1.146	1.161	1.172	1.185	1.198	1.211	1.224	1.237	1.250
$\rho$	0.658	0.649	0.639	0.629	0.618	0.606	0.594	0.586	0.574	0.562	0.549	0.534	0.518	0.500
$\mu$	0.141	0.145	0.150	0.156	0.163	0.170	0.177	0.183	0.189	0.198	0.206	0.216	0.226	0.240
*T*	1.276	–	–	–	–	–	–	–	–	–	–	–	–	–
$\rho$	0.447	–	–	–	–	–	–	–	–	–	–	–	–	–
$\mu$	0.284	–	–	–	–	–	–	–	–	–	–	–	–	–
**LJ16-6**														
*T*	0.988	1.020	1.052	1.084	1.116	1.148	1.180	1.212	1.244	1.252	1.272	1.292	1.312	1.332
$\rho$	0.813	0.801	0.789	0.777	0.764	0.748	0.733	0.717	0.702	0.695	0.684	0.673	0.662	0.650
$\mu$	0.051	0.056	0.060	0.065	0.071	0.077	0.084	0.090	0.098	0.101	0.107	0.111	0.116	0.122
*T*	1.373	1.393	1.413	1.428	1.444	1.460	1.476	1.492	1.508	1.525	1.541	–	–	–
$\rho$	0.624	0.610	0.595	0.584	0.570	0.556	0.540	0.522	0.501	0.475	0.437	–	–	–
$\mu$	0.136	0.142	0.149	0.156	0.163	0.171	0.181	0.194	0.208	0.224	0.255	–	–	–
**Ethane**														
*T*	305	300	290	280	270	260	250	240	230	220	210	200	190	180
$\mu$	109	100	83.9	71.7	62.7	56.6	51.5	46.5	41.6	37.7	34.3	30.5	27.3	24.1
${\tilde{\mu }}$	0.324	0.297	0.250	0.213	0.186	0.168	0.153	0.138	0.124	0.112	0.102	0.091	0.081	0.072
*T*	160	151	141	132	120	110	100	–	–	–	–	–	–	–
$\mu$	17.9	15.4	12.6	10.1	7.27	5.2	3.37	–	–	–	–	–	–	–
${\tilde{\mu }}$	0.053	0.046	0.037	0.030	0.022	0.015	0.010	–	–	–	–	–	–	–

**Figure 2:**
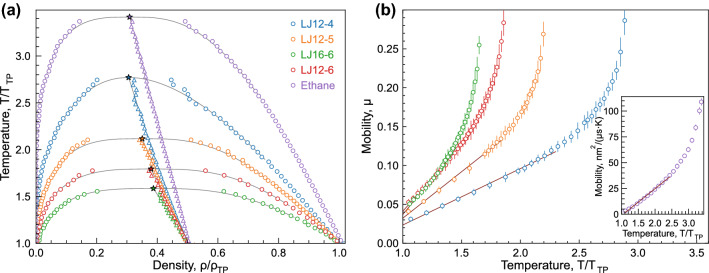
Particle mobility in liquid on gas-liquid binodals: (**a**) liquid–gas binodals of the considered systems. The binodals were calculated by the two-phases simulation method described in Materials and methods section. The color symbol denote the calculated binodals, the triangles denote the median points. The solid gray lines show the temperature range used in approximation and determination of parameters in Eq. (7). (**b**) Temperature dependence of particle mobility in liquid under coexistence with a gas. Straight lines correspond to linear approximations for the mobility. The calculated mobility of ethane is shown as inset in panel (**b**).

The main results of the present paper are shown in Fig. 2, wherein the gas-liquid binodals and the temperature dependencies of particle mobility in considered systems are illustrated in Fig. 2a,b, respectively. The same colours of the symbols in Fig. 2a,b correspond to the same systems. The temperatures and densities are normalised to their values in liquid state at triple point, respectively. In Table 2, the mobility coefficients of generalized LJ liquids at different temperatures on the liquid–gas binodal are tabulated. The temperature and mobility are shown in dimensionless units, using the LJ energy and length scales for normalization. For ethane, the temperature and mobility are provided in kelvins *K* and $nm^2 / (\mu s \cdot K)$, respectively.

The temperatures of the triple and critical points, as well as their ratio $T_{\textrm{CP}}/T_{\textrm{TP}}$, are observed to increase for more long-ranged attraction, as we see in Fig. 2a comparing the results for the series LJ12-4, LJ12-5, and LJ12-6. Comparing the results for LJ16-6 and LJ12-6, we see a weak increase in $T_{\textrm{CP}}$ with increase in repulsion exponent (see Table 1), but also that the ratio $T_{\textrm{CP}}/T_{\textrm{TP}}$ drops, as shown with green symbols in Fig. 2a.

In general case, mobility is a nonlinear function of temperature and pressure, being difficult to be predictable accurately in a broad range of thermodynamic parameters. However, we surprisingly discover that $\mu (T)$ exhibits a linear growth at the liquid branch of the liquid–gas binodal, from the triple point to approximately $0.8T_{\textrm{CP}}$. Moreover, we see in Fig. 2b that this linear behaviour is inherent to all the systems investigated.

The temperature susceptibility of the mobility $\gamma = \partial \mu /\partial T$ increases for potentials with more short-range attraction (larger attractive exponent), as one can see in Fig. 2b. The results for ethane are provided in the inset in Fig. 2b and clearly illustrate the same trend—linear $\mu (T)$-dependence above the triple point and transition (crossover) to the nonlinear regime at some temperature.

### Collective excitations across coupling regimes

**Figure 3:**
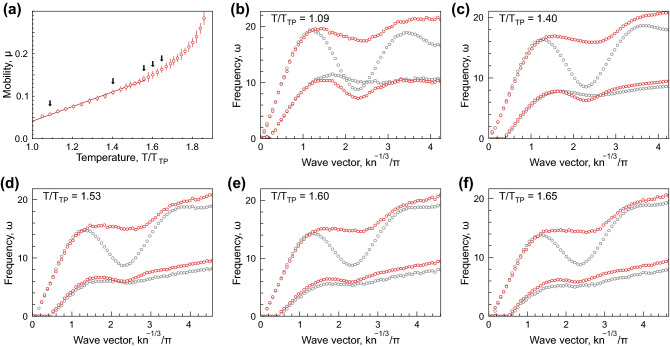
Particle mobility and excitation spectra: (**a**) Temperature dependence of the mobility coefficient in LJ12-6 system at the liquid branch of the gas-liquid binodal. Black arrows indicate the temperatures, at which the excitation spectra shown in panels (**b**)–(**f**) were calculated. (**b**)–(**f**) excitation spectra in LJ12-6 fluid at the liquid–gas binodal; spectra obtained using the single mode (longitudinal and transverse) analysis are shown with gray symbols; spectra obtained using the two-oscillator model (high- and low-frequency hybridised modes) are shown with red symbols. The reduced temperatures are indicated in the upper left corners.

Within the framework of collective excitations, the change in diffusion regime should be attributed to the change in collective excitation spectra. To try this hypothesis in our case, we calculated the spectra in liquids at different points in the gas-liquid binodals (see Methods for details). The examples of spectra obtained for LJ12-6 system are shown in Fig. 3. Here, the particle mobility $\mu (T)$ for LJ12-6 liquid, shown in Fig. 2b, is reproduced in Fig. 3a. Black arrows indicate the temperatures at which the excitation spectra are presented in Figs. 3b–f:Fig. 3b corresponds to the state near the triple point, whereas Fig. 3c—f illustrate evolution of the spectra with an increase in temperature, in the temperature range wherein $\mu (T)$ becomes nonlinear approaching the critical point.

The dispersion relations shown in Fig. 3b–f were obtained using two approaches (extensively discussed in Refs.^39–42^), depending on the method used for the current spectra (8) analysis. Here, the results for separate analysis of excitations with longitudinal and transverse polarisations (single mode analysis^39^) are shown using gray symbols. Within this framework, the spectra $C_{L,T}(q,\omega )$ (8) should be calculated using MD data and then fitted with theoretical models corresponding to a damped harmonic oscillator. After this, we obtain the dispersion relations and damping rates for fluctuations with longitudinal and transverse polarisations (L- and T-modes). In this approach, the conditions at which $\mu (T)$ becomes nonlinear correspond approximately to the change from crossed to uncrossed dispersion curves for $\omega _{L}$ and $\omega _{T}$, as illustrated in Fig. 3b–c.

The results of the analysis using the two-oscillator model, taking into account strong scattering of excitations at large *q* and their possible mixing^40–42^, are shown in Fig. 3b–f with red symbols. Here, the dispersion relations and damping rates are obtained using the two-oscillator model to fit the total current spectra (8), $C(q, \omega )=C_L(q, \omega )+C_T(q, \omega )$, instead of separate mode fitting (see Ref.^39^ for details). In this case, we speak about high- and low-frequency excitation spectra.

Simultaneously with the onset in nonlinear behaviour of $\mu (T)$, the high-frequency dispersion relation changes from an oscillating (solid-like) to a monotonously increasing (gaseous) shape, as illustrated in Fig. 3b–f. Thus, the crossover in $\mu (T)$ behaviour is approximately accompanied by a qualitative change in the excitation spectra. The observed relationships are not specific for the LJ12-6 system only. The same trends are observed for other generalised LJ systems investigated. This is illustrated in Fig. 6–8 provided in Methods.

**Figure 4:**
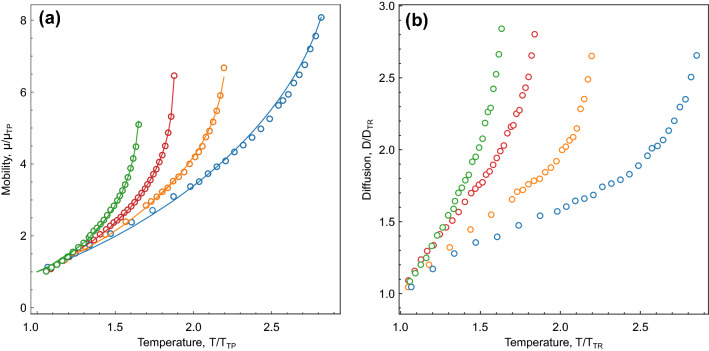
Reduced particle mobility (**a**) and diffusion (**b**) on the liquid branch of the gas-liquid binodal versus the reduced temperature for generalised Lennard–Jones systems: Symbols are MD results and solid lines are fits by 5.

Surprisingly, the linear growth in particle mobility across coupling regimes above the triple point is inherent to all systems we considered. Moreover, we found an empirical fit for dependence of particle mobility on the density and temperature:

5 $$\begin{aligned} \mu = \mu _{\textrm{TP}}\left( \frac{\rho }{\rho _{\textrm{TP}}}\right) ^{-3(T_{\textrm{CP}}/T_{\textrm{TP}})^{3/2}}\left( \frac{T}{T_{\textrm{TP}}}\right) ^{4/3}, \end{aligned}$$

where $\mu _{\textrm{TP}}$ is the mobility at the triple point. The comparison between the MD results and the fit of Eq. (5) is shown in Fig. 4. We observe that the proposed fit describes numerical data quite accurately for a broad range of interactions considered. Equation (5) can also be used to describe MD results in the case of ethane, two-particle molecules, but the exponent in the reduced temperature dependence should be changed to 7/3 (from 4/3). We hope that the reported numerical and empirical results will stimulate further theoretical and experimental studies to understand the reasons leading to the particular scaling $\mu (T)$ we observed.

## Conclusion

In this work, we studied relationships between the diffusion and mobility coefficients in liquids with different pairwise interactions between particles on the one hand and respective excitation spectra of collective modes on the other hand. With MD simulations, we considered systems of particles interacting via a generalised LJ potential with different attractive and repulsive exponents, as well as ethane. We calculated the particle mobility and observed that it increases linearly with temperature above the triple point on liquid branch of the gas-liquid binodal. Analysis of excitation spectra revealed that the onset of non-linear behaviour of $\mu (T)$ corresponds to the crossover in the shape of the dispersion relations of collective excitation spectra. The thermal susceptibility of the mobility, $\partial \mu / \partial T$, expectedly increases for more short-range attraction between particles, because of the particles move less time in the range of potential created by their neighbors.

Diffusion is known to be hard to predict for a broad range of thermodynamical parameters in condensed phases. The correlation between transport properties (particle mobility) and dispersion relations is expected, but has never been observed directly and analyzed in detail. Therefore, a simple linear relationship identified in the present paper provides a new evidence regarding relations between transport and collective excitation properties in liquids.

Direct experimental check of the results can be performed using experiments with colloids with tunable interactions in external rotating electric or magnetic fields. In this case, the liquid state on the binodal between triple and critical point can be realized by changing the magnitude of applied rotating field. Moreover, the interactions in such systems have been shown recently to include essentially many-body component^43–47^, which can be tuned with conically-rotating external fields.

Among prospective problems for future studies, one can notify a study of the effects provided by many-body interactions, more inherent to real systems, compared to simple pairwise interactions considered here. Another problem might be related with detailed theoretical treatment, since the linearity might mean an existence of a small parameter, yet unknown. Finally, a similar study can be performed for active fluids, to identify the relationship between excitations spectra and diffusion in such systems. We believe that the reported results will open novel prospects for corresponding studies of various fluids, from simple to molecular liquids, active and living systems.

## Materials and methods

**Figure 5:**
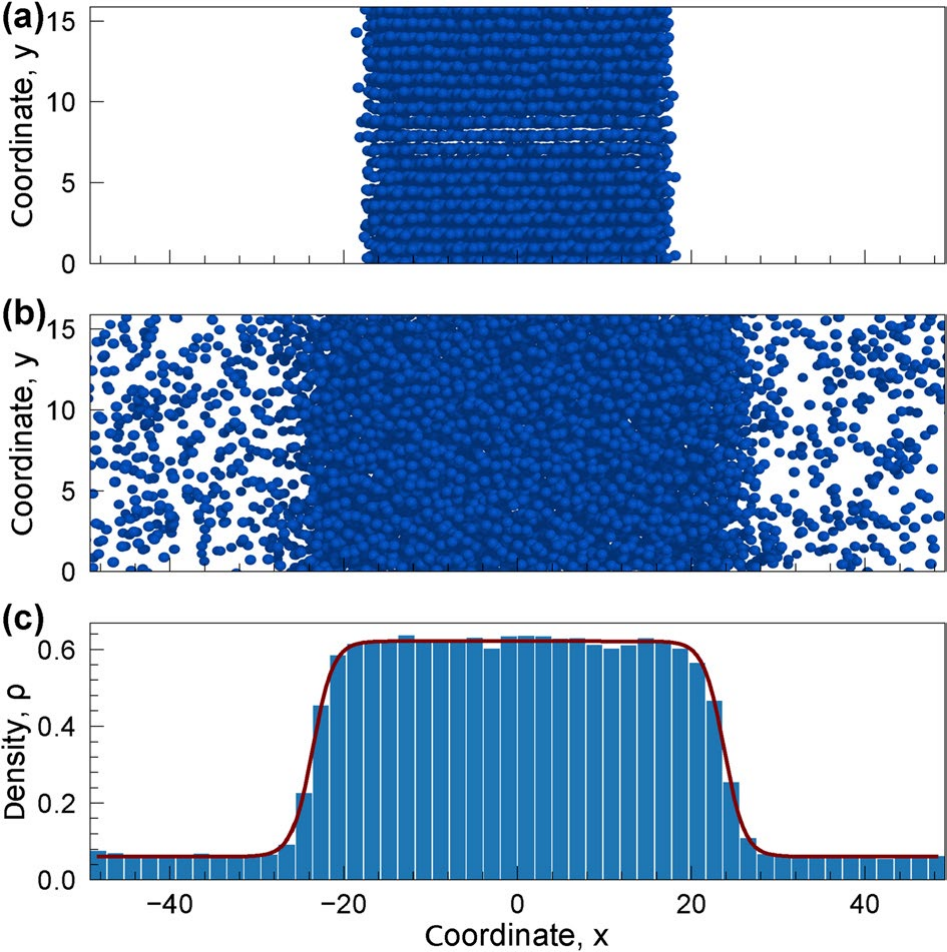
Flat layer of LJ12-6 particles: Snapshot of the system in initial state (**a**) and at $T = 1.13$ (**b**). (**c**) Density profile of the system along the *x*-axis. The high- and low-density regions are in the liquid and gas state, respectively. The dark red line is the approximation of the density profile by Eq. (6).

All MD simulations were performed in the *NVT* ensemble (*N*, *V*, and *T* are number of particles, volume, and temperature of the system, respectively) with help of Nose-Hoover thermostat and periodic boundary conditions using LAMMPS simulation package^48^. At the first step, we calculated binodal lines following Ref.^49^. The initial state of the system was prepared in two steps: (i) the cubic simulation box was filled by fcc crystal of *N* particles with the number density corresponding to near zero pressure; (ii) the simulation box was expanded in the *x*-axis direction so that the final average density of the system $\rho$ became equal to the values specified in Table 3. Here, $r_c$ is the cutoff radius, $T_{\textrm{start}}$ and $T_{\textrm{stop}}$ are the initial and final temperatures of the simulation, respectively, $n_{\textrm{step}}$ is the number of simulation steps, and $\Delta t$ is the time step. The cutoff radii $r_c$ for LJ$n-m$ potentials were chosen to provide $U_{n-m}(r_c)/\varepsilon$ is at least less than $10^{-4}$. The initial state is illustrated in Fig. 5a. Then, the temperature was linearly increased from $T_{\textrm{start}}$ to $T_{\textrm{stop}}$ over $n_{\textrm{step}}$ simulation steps with the time step $\Delta t$. The condensed phase started to evaporate at some moment, forming a coexistence of gas and condensate at the temperature below the critical one, as illustrated in Fig. 5b. Crucial that a system state obtained by this way almost always had phase boundaries orthogonal to the *x*-axis. As a result, densities $\rho _g$ and $\rho _c$ of gaseous and condensed phases, respectively, can be calculated through the fitting of density profile $\rho (x)$ by the expression^49^:

6 $$\begin{aligned} \rho (x)=\frac{\rho _{l}+\rho _{g}}{2}-\frac{\rho _{l}-\rho _{g}}{2} \tanh \left( \frac{|x|-L}{\delta }\right) , \end{aligned}$$

where *L* is a half of the area occupied by the liquid phase and $\delta$ is a characteristic width of the interface. An example of the density profile and its approximation by Eq. (6) is shown in Fig. 5c by histogram and red line, respectively. The simulation parameters for considered models are summarized in Table. 3.

**Table 3:** Parameters used in MD simulations for the calculation of the gas-liquid binodals.

Potential	$\rho$	$r_c$	$T_{\textrm{start}}$	$T_{\textrm{stop}}$	$n_{\textrm{step}}$	$\Delta t$
**Dimensionless units**						
LJ12-4	0.33	15.0	1.0	5.5	$3 \times 10^6$	$5 \times 10 ^ {- 4}$
LJ12-5	0.33	10.0	0.8	2.4		
LJ12-6	0.33	8.0	0.5	1.4		
LJ16-6	0.33	8.0	0.8	1.6		
**Dimensional units**						
Ethane	$0.22\mathrm {{g}/{cm^3}}$	$25\mathrm {\text{\AA }}$	$80\,\textrm{K}$	$320\,\textrm{K}$	$2 \times 10^6$	$2\,\textrm{fs}$

In the vicinity of the “liquid–gas” critical point, calculating the densities of the gas and liquid phases becomes difficult because of enhanced density fluctuations. However, the position of the critical point on the phase diagram can be calculated by approximating the liquid and gaseous binodal branches near the critical point by the fit:

7 $$\begin{aligned} \rho _{l}-\rho _{g} \simeq A \tau ^{\beta }, \quad \rho _{l}+\rho _{g} \simeq a \tau +2 \rho _{\textrm{CP}}, \end{aligned}$$

where $\tau =T_{\textrm{CP}}-T$, $T_{\textrm{CP}}$ and $\rho _{\textrm{CP}}$ are the temperature and density at the critical point, $\beta$ is the critical index, *A* and *a* are free parameters. The critical index $\beta$ depends on the system universality class, which is determined by interparticle interaction mechanisms^50^. In 3D systems, the critical index is $\beta = 1/2$ for LJ$12-4$ potential, while $\beta = 1/8$ for LJ12-5, LJ12-6, LJ16-6, and ethane, according to the previous results^36,50,51^.

We calculated the mobility using the densities and temperatures taken from the obtained parameters on the binodals. For generalised LJ systems with $N=4.0\times 10^3$ particles, we perform simulations for $1.5 \times 10^6$ time steps. For ethane, we used $N = 1.065 \times 10^4$ molecules and performed simulations for $7.0 \times 10^5$ time steps. The first $5.0 \times 10^5$ time steps for generalised LJ systems and $5.0 \times 10^5$ time steps for ethane were used for system relaxation. The remaining parameters were the same as those we used for calculating the gas-liquid binodals.

The excitation spectra were obtained using the analysis of the velocity current^39^:

8 $$\begin{aligned} C_{L, T}({\textbf{q}}, \omega )=\int dt e^{i \omega t} \text {Re} \left\langle {\textbf{j}}_{L, T}({\textbf{q}}, t) {\textbf{j}}_{L, T}(-{\textbf{q}}, 0)\right\rangle , \end{aligned}$$

where ${\textbf{q}}$ and $\omega$ are the wave vector and the frequency, ${\textbf{j}}_{L}={\textbf{q}}({\textbf{j}} \cdot {\textbf{q}}) / q^{2}$ and ${\textbf{j}}_{T}=(\mathbf {j \cdot e_{\perp })e_{\perp }}$ are the longitudinal (*L*) and transverse (*T*) components of the particle current ${\textbf{j}}({\textbf{q}}, t)=N^{-1} \sum _{s} {\textbf{v}}_{s}(t) \exp \left( i {\textbf{q}} {\textbf{r}}_{s}(t)\right)$, and ${\textbf{v}}_{s}(t)=\dot{{\textbf{r}}}_{s}(t)$ is the velocity of the *s*th particle. The summation is over all *N* particles in the system. Averaging over the canonical ensemble is denoted by $\langle \cdots \rangle$. The analysis of $C_{L, T}({\textbf{q}}, \omega )$ was performed according to the procedures described in Ref.^39–42^, enabling to obtain the excitation spectra of high- and low-frequency modes, as well as for longitudinal and transverse excitations separately, using analysis of the velocity current (8).

**Figure 6:**
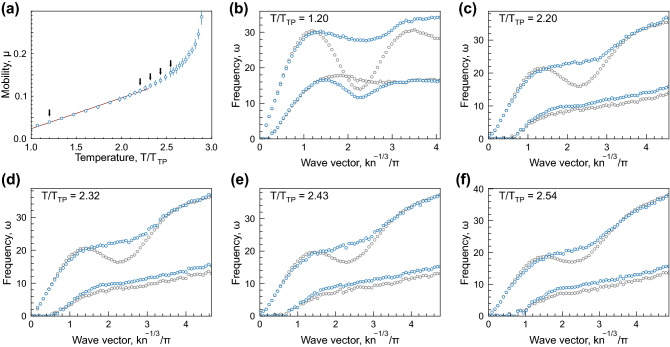
Particle mobility and dispersion relations: The results for LJ12-4 potential, description is the same as in Fig. 3.

**Figure 7:**
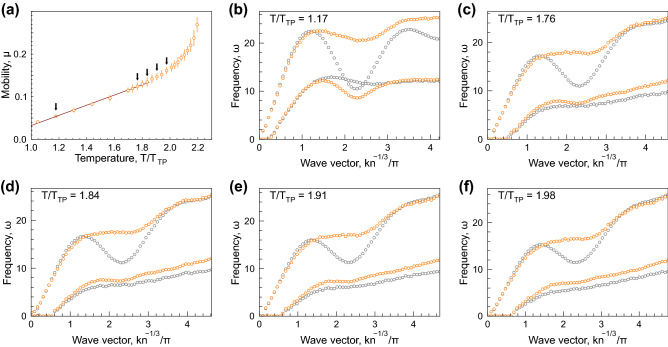
Particle mobility and dispersion relations: The results for LJ12-5 potential, description is the same as in Fig. 3.

**Figure 8:**
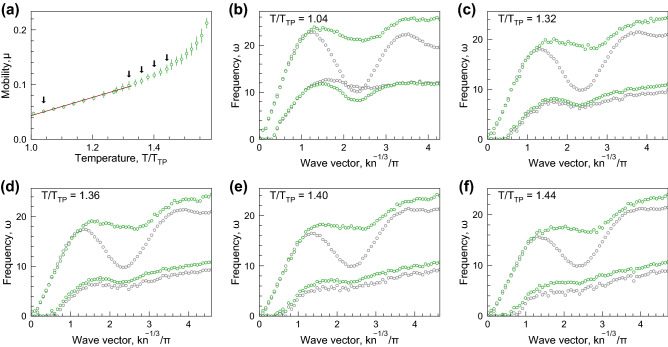
Particle mobility and dispersion relations: The results for LJ16-6 potential, description is the same as in Fig. 3.

MD simulation for the calculation of the excitation spectra differs from that for the mobility only by the duration of the time step. For LJ12-4 and LJ16-6, the time step was chosen as $\Delta t = 1 \times 10^{-4} \sqrt{m\sigma ^2/\varepsilon }$, while the time step for LJ12-5 and LJ12-6 was $\Delta t = 5 \times 10^{-4} \sqrt{m\sigma ^2/\varepsilon }$.

Particle mobility and excitation spectra are shown in Figs. 6–8 for LJ12-4, LJ12-5, and LJ16-6 systems. The description of the panels is the same as that for Fig. 3.

## Data Availability

The datasets used and/or analysed during the current study available from the corresponding author on reasonable request.
